# Isolated cerebellar metastasis from urothelial carcinoma: A case report of a rare phenomenon

**DOI:** 10.1016/j.bas.2023.102716

**Published:** 2023-11-29

**Authors:** Eduard J.A. Verheijen, Walter Taal, Rishi D.S. Nandoe Tewari, Mariëtte C.A. Giessen, Hossain Roshani

**Affiliations:** a Departments of Urology, Haga Teaching Hospital, The Hague, the Netherlands; b Neurology, Haga Teaching Hospital, The Hague, the Netherlands; c Departments of Neurosurgery, Haaglanden Medical Centre, The Hague, the Netherlands; d Pathology, Haaglanden Medical Centre, The Hague, the Netherlands; e Department of Neurology/Neuro-Oncology, Erasmus MC Cancer Institute, Rotterdam, the Netherlands

**Keywords:** Urothelial carcinoma, Isolated cerebellar metastasis, Central nervous system, Tumor resection, Radiotherapy

## Abstract

**Introduction:**

Although urothelial carcinoma (UC) generally is non-invasive, contrastingly in 25% of patients UC metastasizes. Isolated central nervous system (CNS) metastasis from UC without other distant metastases are considered rare. In this report we describe a patient with an isolated and solitary cerebellar metastasis from UC.

**Research question:**

In this case report we explore the value of histological analysis of CNS metastases, imaging, treatment options and survival.

**Material and methods:**

A rare case is presented of a patient diagnosed with an isolated CNS metastasis originating from UC. Through a systematic review of literature route of dissemination, current imaging and treatment options, and survival are discussed.

**Results:**

A 77-year-old male was diagnosed with a pT2N0M0 high-grade UC and treated with transurethral resection and chemoradiation therapy. Several months later, the patient presented with neurological symptoms, and radiological imaging revealed a solitary cerebellar mass. A body CT scan showed no other metastasis. After surgical resection, histology confirmed urothelial origin of the mass, matching his primary UC and the patient received post-operative stereotactic radiotherapy at the surgical site. Recurrence of the cerebellar mass occurred after 6 months for which the patient received re-resection. The patient died 5.5 months after re-resection.

**Discussion and conclusion:**

Isolated brain metastases without other distant metastases from UC are rare, so histologic confirmation of the brain metastasis is essential, particularly when the time interval between diagnosis of the UC and brain metastasis increases. Early brain CT is not recommended. PET CT may have additional value in detection of other distant metastases from UC. Despite advancements in treatments, prognosis for CNS metastasis from UC remains poor.

## Highlights

• Isolated CNS metastases from primary urothelial carcinoma are rare.
• Histological confirmation of the metastatic origin is key.
• Despite advancements in treatments, prognosis remains poor.

## 1 Introduction

Urothelial carcinoma (UC) is the most common type of lower urinary tract cancer comprising approximately 90% of all bladder tumours (Wong et al., 2018). The annual incidence is estimated to be 393.000 patients worldwide with 5500 cases per year in the Netherlands. The incidence increases with age and the majority of patients are above 65 years old. Several risk factors have been identified for development of UC including smoking, exposure to carcinogenic agents, chronic cystitis, HPV infection and a history of radiotherapy or cyclophosphamide treatment (Ebrahimi et al., 2019; Nederland, 2021; Burger et al., 2013; Daneshmand et al., 2021). Although most UCs (non-muscle invasive bladder cancer; NMIBC) are limited to the mucosa or submucosa, approximately 25% progress to muscle invasive bladder cancer and metastasize (Bellmunt et al., 2021).

Metastases from UC are predominantly found in peri-vesical lymph nodes (70%), bones (49%), lungs (35%) and liver (27%) (Shinagare et al., 2011). Central nervous system (CNS) metastases from UC are rare as most CNS metastases originate from lung-, breast-, colon-, or kidney cancer, or melanoma (Barnholtz-Sloan et al., 2004). Since brain metastases with urothelial origin occur very rarely routine imaging for metastatic spread of UC covers the thoracic and abdominal cavities but not the cranial region and, therefore, these metastases are usually only detected after the patient presents with neurological symptoms. Furthermore, CNS metastasis from UC without other distant metastases occurs even less frequently and the number of published papers covering this phenomenon is in a total of 36 patients (Anderson et al., 1992; Bruna et al., 2003; Butchart et al., 2010; Clatterbuck et al., 1998; Cozzarini et al., 1999; Crowley et al., 2008; D’Souza et al., 2011; Davies et al., 2003; Davis et al., 1986; Diamantopoulos et al., 2020; Erhamamcı et al., 2014; Findler et al., 1983; Gardner, 2013; Girgis et al., 1999; Hayashi et al., 2011; Kabalin et al., 1988; Kartha et al., 2015; Kuppa et al., 2021; Leadbetter and Colston, 1937; Mahmoud-Ahmed et al., 2002; Majcherczyk et al., 2021; Qasho et al., 1999; Salvati et al., 1993; Santarossa et al., 1997; Shamdas et al., 1992; Stastny et al., 1996; Worm et al., 2016; Zennami et al., 2008). In this case report we present a patient with an isolated cerebellar metastasis and no signs of extracranial disease activity, originating from his primary UC.

## 2 Case presentation

### 2.1 Clinical presentation

A 77-year-old male presented at the emergency department in August 2020 with complaints of haematuria since several hours. He had a medical history of chronic renal disease diagnosed in December 2019 that was preceded by an episode of macroscopic haematuria with urinary retention for which he was catheterized. The patient had not recently experienced any urinary tract infections nor used any anticoagulant medication. Upon presentation at the emergency department, the bladder was irrigated to clear urine and the patient was discharged upon further outpatient examinations.

Two weeks later CT Intravenous Pyelography (CT IVP) was performed which demonstrated a diffusely irregularly thickened wall of the bladder on the cranioventral side. Cystoscopy revealed no evident irregularities at that time. Transurethral resection of the suspected lesion of the bladder wall was performed and pathological analysis revealed a urothelial carcinoma reaching into the musculus detrusor layer. Additional CT imaging of the thoracic and abdominal regions did not demonstrate any further metastases; therefore, the tumour was classified as a pT2N0M0 high grade carcinoma.

Although surgical treatment through radical cystectomy with Bricker ileal conduit was considered possible, the patient opted for chemoradiation therapy as advised during a multidisciplinary consultation meeting due to his impaired renal function. The patient received radiotherapy with an elective nodal dose of 50 Gy and 63.25 Gy aimed at the primary tumour divided over five fractions per week for the duration of five weeks. Concurrently, the patient started chemotherapy through a single treatment with mitomycin and daily dose of capecitabine during the same five weeks. At four months follow-up, CT thorax, abdomen and cystoscopy revealed complete response and no metastases.

Eight months after the primary diagnosis, the patient presented at the emergency department with neck pain radiating towards the left side of his head. He had vomited and required support during walking due to ataxia. The patient was admitted for further diagnostic work-up.

### 2.2 Imaging

**Fig. 1:**
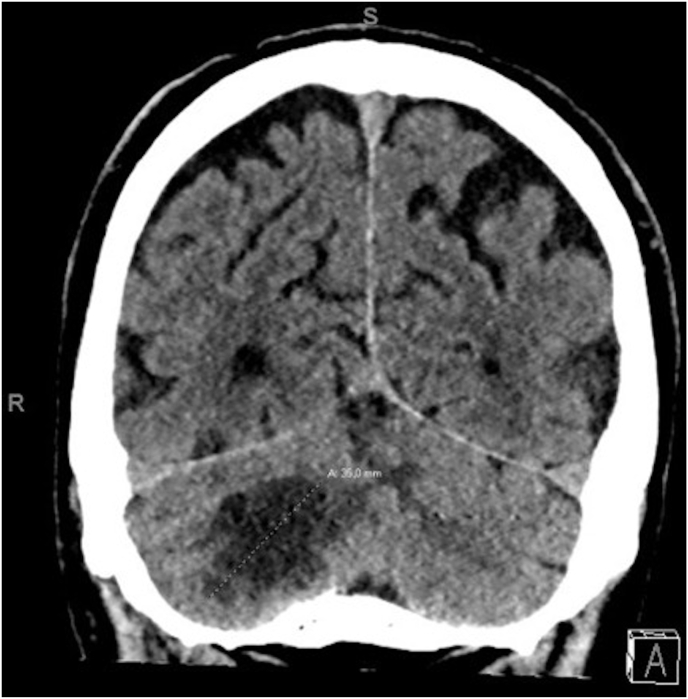
Coronal view of brain CT imaging demonstrating a solitary, partially necrotic lesion in the right cerebellar hemisphere. The maximum diameter of the cerebellar lesion measured 35.0 mm.

**Fig. 2:**
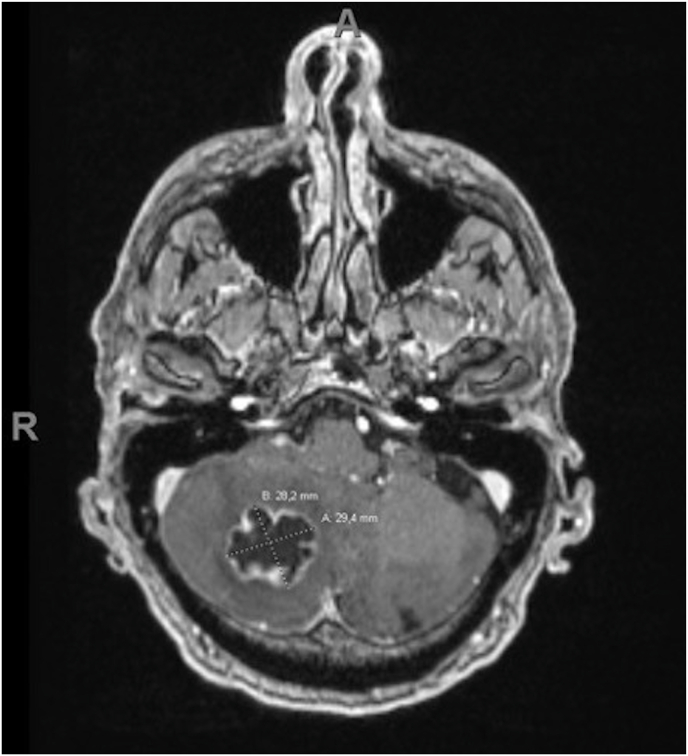
T1-weighted gadolinium-enhanced transverse image of MRI brain examination. The metastatic lesion is located in the right cerebellar hemisphere obstructing the fourth ventricle without supratentorial hydrocephalus.

A CT and MRI scan of the brain showed a solitary 29 mm in diameter cerebellar mass in the right hemisphere with some hypodensities indicating partial necrosis of the lesion. Furthermore, due to tumour oedema a slight midline shifting causing compression of the brain stem and fourth ventricle was observed (Fig. 1, Fig. 2). Repeated thoracic and abdominal CT imaging did not demonstrate any other primary malignancy nor metastatic lesions of the primary tumour.

### 2.3 Therapeutic approach

After neurosurgical consultation, the cerebellar mass was surgically removed through a lateral suboccipital craniotomy. During surgery, the cerebellum first appeared swollen due to the mass, however quickly relaxed after opening of the cystic cavity and cerebrospinal fluid drainage. The tumour could be completely resected, using an ultrasonic aspirator (CUSA) with a small margin around the tumour. The dura was primarily closed, and the bone flap returned. Navigation was used during surgery to control the extend of resection and one day postoperatively confirmed on MRI finding. Postoperatively, dexamethasone could be stopped completely, and the patient was discharged two days after surgery from the hospital.

### 2.4 Histological findings

During surgery, the samples had a brown-grey appearance with a lamellar structure, partially haemorrhagic. Histological analysis revealed an epithelial tumour featuring irregularly enlarged and partially polymorphous nuclei and substantial cytoplasm. Immunohistochemistry revealed that the tumour cells were positive for keratin 5/6, keratin 7, keratin 20, keratin 34 beta E12 and GATA-3. All histological findings matched the report of the primary urothelial origin (Fig. 3a, Fig. 3b).

**Fig. 3a:**
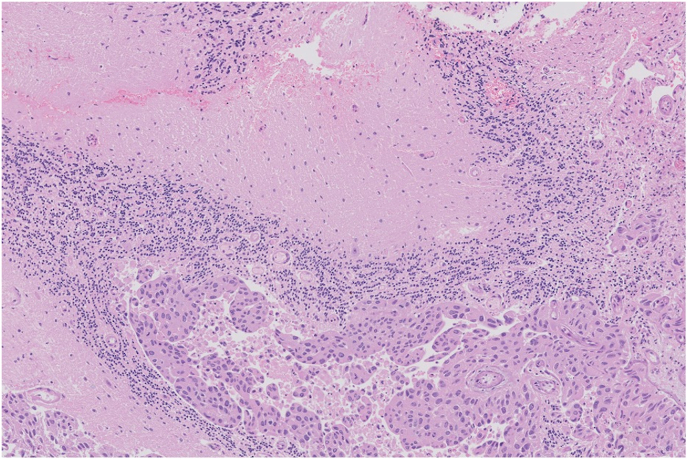
Microscopic haematoxylin-eosin staining of the cerebellar lesion. In the upper part normal cerebellar cortex is visualized. In the lower part atypical cells/tumour cells arranged in epithelial clusters can be recognized.

**Fig. 3b:**
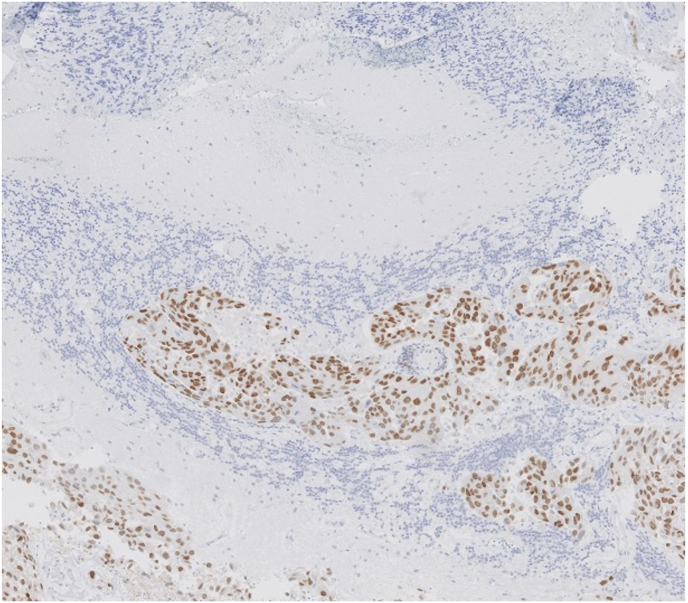
Microscopic staining with GATA-3. The tumour cells are coloured brown since their nuclei are positive for this marker confirming urothelial origin. (For interpretation of the references to colour in this figure legend, the reader is referred to the Web version of this article.)

### 2.5 Post-operative follow-up

After treatment, the patient fully recovered and regained full physical functionality. Follow-up MRI examination one day after surgery showed a complete resection. The patient started post-operative stereotactic radiotherapy one month after surgery. A single dose of 18 Gy was administered to the resection cavity and surrounding cerebellar tissue.

Repeat cystoscopy 12 and 18 months after initial presentation demonstrated no signs of recurrence of UC in the bladder. Additionally, at 18 months follow-up the patient underwent CT examination of the thoracic and abdominal regions which demonstrated no metastatic activity in the body. However, seven months after the first surgery, the patient showed recurrent signs of ataxia and MRI examination of the brain revealed a contrast enhancement without increased perfusion. Since it is clinically and radiologically difficult to distinguish recurrence from necrosis after SRS, we opted for early repeat MRI in a wait-and-scan matter. Radiological follow-up six weeks later, showed growth of the lesion with oedema and compression of the fourth ventricle and radiological signs of supratentorial hydrocephalus. Because of the mass effect and growing suspect of progressive disease, surgical decompression was discussed with the patient, and was opted for second surgery. The patient underwent re-resection of the lesion two weeks later and histological analysis confirmed metastatic recurrence with partially vital and partially necrotic tumorous tissue. The patient was referred for a second session of post-operative stereotactic radiotherapy. The patient died 5.5 months after the re-operation.

## 3 Discussion

Brain metastases account for 13–39% of all intracranial malignancies and are often detected at a late stage of systemic malignancy (Haberland, 2007; Moes and Vogel, 2009; Walker and Kapoor, 2007). The majority of brain metastases are located in the supratentorial region, however, 15% is found in the cerebellar hemispheres (Fink and Fink, 2013). In general, the prognosis after brain metastasis is poor and among others depends on the type of primary malignancy with an overall median survival of 13 months (Sperduto et al., 2020). Brain metastases usually originate from primary tumours in the lungs (19.9%), skin (6.9%), kidneys (6.5%), breasts (5.1%) and gastrointestinal tract (1.9%) (Barnholtz-Sloan et al., 2004). In this case report we present a patient with a solitary metastasis of his primary UC.

Isolated brain metastases (i.e., without other distant metastases) from UC are rare. UC tends to metastasize first to thoracic or abdominal structures and from these sites to the central nervous system in up to 7% of patients with bladder cancer (Clatterbuck et al., 1998). However, the incidence of CNS metastases from urothelial origin has been increasing over the years. In general, with the introduction of targeted therapies and immunotherapies, the survival of patients with cancer has prolonged, and more CNS metastases in this course are observed (Sarmiento et al., 2012). Moreover, improvements in radiological imaging techniques, may have resulted in a further increase of CNS metastases detection. Furthermore, chemotherapy remains the standard treatment strategy for distant metastases of UC, however, its inability to pass the blood brain barrier could make it less effective for CNS metastases resulting in an increasing incidence of these lesions (Davies et al., 2003).

Only a limited number of case reports and case series has been published on histologically-confirmed isolated CNS metastasis without other distant metastases from UC in a total of 36 patients (Anderson et al., 1992; Bruna et al., 2003; Butchart et al., 2010; Clatterbuck et al., 1998; Cozzarini et al., 1999; Crowley et al., 2008; D’Souza et al., 2011; Davies et al., 2003; Davis et al., 1986; Diamantopoulos et al., 2020; Erhamamcı et al., 2014; Findler et al., 1983; Gardner, 2013; Girgis et al., 1999; Hayashi et al., 2011; Kabalin et al., 1988; Kartha et al., 2015; Kuppa et al., 2021; Leadbetter and Colston, 1937; Mahmoud-Ahmed et al., 2002; Majcherczyk et al., 2021; Qasho et al., 1999; Salvati et al., 1993; Santarossa et al., 1997; Shamdas et al., 1992; Stastny et al., 1996; Worm et al., 2016; Zennami et al., 2008) (Table 1). In general, these CNS metastases are only identified when the patient presents with neurological symptoms, as routine MRI scans of the brain are usually not done in UC patients. In literature, the median time to detection of isolated CNS metastasis after UC diagnosis is 6 months (range 0–42 months) (Anderson et al., 1992; Bruna et al., 2003; Clatterbuck et al., 1998; Cozzarini et al., 1999; Crowley et al., 2008; D’Souza et al., 2011; Davies et al., 2003; Davis et al., 1986; Diamantopoulos et al., 2020; Erhamamcı et al., 2014; Findler et al., 1983; Girgis et al., 1999; Hayashi et al., 2011; Kabalin et al., 1988; Kartha et al., 2015; Kuppa et al., 2021; Leadbetter and Colston, 1937; Mahmoud-Ahmed et al., 2002; Majcherczyk et al., 2021; Qasho et al., 1999; Salvati et al., 1993; Shamdas et al., 1992; Stastny et al., 1996; Worm et al., 2016; Zennami et al., 2008). Several other articles describe CNS metastasis from UC, but in these reports extracranial metastases preceded or were concurrently identified with CNS metastasis, or the urothelial origin of the CNS metastasis was not confirmed by histological analysis (Chandra et al., 2020; Ogunbona et al., 2021; Perlmutter et al., 2006; Vaa et al., 2014; Zigouris et al., 2009). Without histological confirmation clinicians should consider other differential diagnoses including a primary CNS tumour or metastasis from another primary site than UC (Kim and Feiden, 2007).

**Table 1:** Overview of literature on isolated, histologically confirmed CNS metastasis from UC without other distant metastases.

Authors, year (reference)	Article type	Number of patients	Gender/age	Stage primary tumour	CNS location	Solitary/multiple metastases	Interval between primary and metastasis	Neurologic deficits	Treatment	Survival
Anderson et al., 1992 [9]	Case series	3	M 62	D2	Brain	Solitary	Concurrent	–	Resection + radiotherapy	14 mo
			M 54	C	Brain	Solitary	23 mo	–	Resection + radiotherapy	5 yr
			M 62	C	Brain	Solitary	42 mo	–	Resection + radiotherapy	9 mo
Bruna et al., 2003 [10]	Case report	1	M 66	pT3_a_N_x_M1	Leptomeningeal	–	Concurrent	Seizures	Intrathecal chemotherapy	2 mo
Butchart et al., 2010 [11]	Case report	1	M 58	T2N1M0	Leptomeningeal	–	>5 mo	Headache, ataxia, vomiting	–	1 mo
Clatterbuck et al., 1998 [12]	Case report	1	M 72	Stage I	Cerebral, left	Solitary	Concurrent	Right homonymous hemianopsia, Gerstmann’s syndrome	Decadron, resection, chemotherapy, WBRT	11 mo
Cozzarini et al., 1999 [13]	Case series	1	M 40	pT3G3pN0	Leptomeningeal	–	10 mo	Headache, lethargy, confusion, 7th cranial nerve palsy, impaired vision, weakness of right extremities	Intrathecal methotrexate	>1 wk
Crowley et al., 2008 [14]	Case report	1	M 74	High-grade invading the perivesicular tissue	Intramedullary, T1-T2	Solitary	6 mo	Brown-Séquard syndrome	Subtotal resection, external beam radiation therapy	2 mo
D’Souza et al., 2011 [15]	Case report	1	F 69	G3pT1	Cerebellar, right hemisphere	Solitary	11 mo	Slurred speech, left-sided facial drooping, left eye visual disturbance, ataxia, nausea, confusion, headache	Resection, WBRT	>21 mo
Davies et al., 2003 [16]	Case report	1	M 56	T1	Cerebellar, right hemisphere	Solitary	Concurrent	Headache, ataxia	Resection, stereotactic radiotherapy, chemotherapy	–
Davis et al., 1986 [17]	Case report	1	M 54	Grade III, stage B2	Cerebral, left	Solitary	5 mo	Focal seizures, lethargy, disorientation, aphasia	Dexamethasone, phenytoin, WBRT, resection, chemotherapy	>18 mo
Diamantopoulos et al., 2020 [18]	Retrospective cohort	1	? 45	T2	Cerebral	Multiple	11.8 mo	–	WBRT, resection	6 mo
Erhamamcı et al., 2014 [19]	Case report	1	M 45	T2N0	Cerebral	Multiple	11 mo	Nausea, dizziness, loss of balance	Excisional biopsy, whole brain radiotherapy	16 mo
Findler et al., 1983 [20]	Case report	1	M 67	–	Cerebral, right hemisphere	Solitary	2 yr	Sudden onset of confabulation, confusion, and memory loss	Biopsy + radiotherapy	–
Gardner, 2013 [21]	Case report	1	M 74	Grade IV, T4bN2M0	Cerebellar, left vermis	Solitary	>11 mo	Unstable gait, fatigue, dizziness, disequilibrium, diplopia	Resection, WBRT	>9 mo
Girgis et al., 1999 [22]	Case report	1	M 72	–	Cerebral, right hemisphere	Solitary	5 mo	Changed behaviour, left facial drooping, diminished motor skills	Resection	9 mo
Hayashi et al., 2011 [23]	Case series	1	M 76	pT1N0M1	Cerebral, left parietal	Solitary	Concurrent	Dysphasia	Resection	>8 yr
Kabalin et al., 1988 [24]	Case series	2	F 62	Grade III/IV	Cerebellar, right hemisphere	Solitary	12 mo	Nausea, vomiting, confusion	None	Postmortem
			M74	Grade III/IV	Cerebellar, left hemisphere	Solitary	4 mo	Nausea, vertigo, headaches	Resection	2 mo
Kartha et al., 2015 [25]	Case report	1	M 59	Grade III	Cerebellar, right vermis	Solitary	Concurrent	Increasing headache	Resection + WBRT	11 yr
Kuppa et al., 2021 [26]	Case report	1	M 58	ypT0	Left cerebellum, right frontal	Multiple	15 mo	Nausea, vomiting, dizziness, weakness and urinary incontinence	Resection (cerebellum) + SRS (cerebral laesions) + radiation therapy + immunotherapy	>23 mo
Leadbetter and Colston, 1937 [27]	Case report	1	M 47	Grade IV	Cerebral, left frontal	Solitary	3 mo	Weakness/unsteadiness right leg, headaches, partial visual impairment, vomiting, drowsiness	Resection	0 d^a^
Mahmoud-Ahmed et al., 2002 [28]	Case series	1	F 69	T4aN1M0	Cerebral, frontal	Multiple	0.5 mo	Seizures	Resection	1.25 mo
Majcherczyk et al., 2021 [29]	Case report	1	M 55	T2 high-grade	Cerebellum, both hemispheres	Solitary	Concurrent	Headache, dizziness, unilateral ataxia	Antiedema treatment, resection, radiotherapy, chemotherapy	–
Qasho et al., 1999 [30]	Case report	1	M 40	Bladder wall laesion	Choroid plexus right lateral plexus	Solitary	Concurrent	Seizures, temporo-spatial disorientation, impairment of long-term memory	Resection	–
Salvati et al., 1993 [31]	Case series	6	M 55	b2/III	Cerebral, right frontal	Solitary	5.1 mo	Intracranial hypertension	Surgery + WBRT	9.2 mo
			F 57	c/II	Cerebral, right temporal	Solitary	8.1 mo	Inracranial hypertension, seizures	Surgery + WBRT	6 mo
			M 63	d1/III	Cerebral, left frontal	Solitary	3.1 mo	Intracranial hypertension	Surgery + WBRT	4 mo
			F 71	b2/II	Cerebral, right frontal	Solitary	7.1 mo	Intracranial hypertension, seizures	Surgery + WBRT	7.1 mo
			M 72	c/III	Cerebral, left temporal	Solitary	6 mo	Intracranial hypertension	Surgery + WBRT	9.8 mo
			M 72	c/III	Cerebral, right parietal	Solitary	6 mo	Intracranial hypertension	Surgery + WBRT	7.9 mo
Santarossa et al., 1997 [32]	Case report	1	F 52	T4-N3-M0	Leptomeningeal	–	>8 mo	Headache, loss of memory, confusion, left hemiplegia, episodic seizures	Intrathecal methotrexate + dexamethasone, WBRT	9 mo
Shamdas et al., 1992 [33]	Case report	1	F 84	Grade II TCC with invasion of the smooth muscle wall	Cerebellar, right hemisphere	Solitary	5 mo	Progressive ataxic gait, finger-to-nose and heel-to-knee impairment on right side, acute onset of occipital headaches, mild impairment of recent memory, horizontal nystagmus	Dexamethasone + resection + postoperative external beam radiation	–
Stastny et al., 1996 [34]	Case series	1	F 60	TCC invading the underlying muscularis	Leptomeningeal	–	17 mo	Persistent headaches	Ommaya reservoir, intrathecal methotrexate, cranial radiation therapy	–
Worm et al., 2016 [35]	Case report	1	F 72	–	Cerebellar, vermis	Solitary	2 yr	Headache, dizziness, loss of balance, dysmetria, dysdiadochokinesia	Resection, ventricle-peritoneal bypass	20 mo
Zennami et al., 2008 [36]	Case report	1	M 65	pT1, G3	Cerebral, left parietal	Solitary	34 mo	Headache, homonymous right hemianopia	Surgery	2.6 mo

a Patient died intraoperatively.

Survival of UC CNS metastasis ranges from several days to more than 10 years with most studies reporting a median survival of less than one year depending on the type of treatment (local, systemic or combination) and strongly declines in patients without any intervention (Mahmoud-Ahmed et al., 2002; Protzel et al., 2002; Boyle et al., 2012; Brenneman et al., 2020; Rosenstein et al., 1993). However, the majority of patients included in these studies had multiple CNS metastases and/or simultaneously suffered from extracranial disease activity. Currently, treatment paradigms for newly diagnosed brain metastases involve surgical resection, stereotactic radiosurgery (SRS), whole-brain radiotherapy (WBRT) and/or systemic therapy (Nahed et al., 2019) and should be decided on an individual patient basis in a multidisciplinary setting. For brain metastases from UC, a small retrospective study showed that a combination of surgery and post-operative radiotherapy results in better survival than radiation therapy alone (mean survival time, respectively, 19 and 6 months), although the latter group included patients with multiple CNS metastases (Rosenstein et al., 1993). Furthermore, two randomized-controlled trials have demonstrated that surgery combined with radiotherapy resulted in longer survival, less disease recurrence and superior quality of life compared to radiation therapy only (Patchell et al., 1990; Noordijk et al., 1994). Nevertheless, the patients in these trials suffered from CNS metastases not exclusively originating from UC. Despite the ongoing development in therapeutic options, brain metastases recur in up to 50% of surviving patients within 6–12 months after initial treatment which is also shown in this case report (Loeffler et al., 2022).

Theoretically, early brain CT or MRI for the purpose of CNS metastasis detection in asymptomatic patients could prevent development of neurological symptoms or prolong survival (Niwińska et al., 2010). However, clear evidence to substantiate improved patient outcome and survival due to pre-treatment brain metastasis screening is lacking. In addition, the incidence of CNS metastasis in patients with metastasized UCC is low compared to other primary malignancies, for example non-small lung cancer, which mandates brain MRI during pre-treatment evaluation for certain stages. Moreover, the incidence becomes even lower in the absence of metastatic spread to thoracic or abdominal/pelvic sites and would require a high number needed to screen (NNS) (Cagney et al., 2017; National Comprehensive Cancer Network (NCCN), 2023a). Brain metastases may also develop at a later stage or be too small to be detected requiring repeat imaging during follow-up. As a result, current guidelines on the treatment of UCC only advise routine brain MRI or CT evaluation if the patient has neurological symptoms or is high-risk (e.g., small cell histology), or can be considered if metastatic spread to other sites has been demonstrated (National Comprehensive Cancer Network (NCCN), 2023b). For our patient, it is impossible to conclude whether early brain imaging would have improved outcome as it is unknown when the CNS metastasis started to develop and became distinguishable on imaging.

Several ways of hematogenous spread have been proposed to explain the presence of isolated brain metastasis from UC in our patient. Tumour cells could enter the venous system and directly reach the arterial blood circulation and, subsequently, the cerebellum: (1) via the pulmonary capillaries, or (2) through a paradoxical tumour embolus via a patent foramen ovale. In addition, metastatic distribution by a retrograde venous route via the paravertebral venous plexus allows for direct flow from the venous system towards the cerebellum (Fervenza et al., 2000; Oeppen and Tung, 2001; Batson, 1940; Morita et al., 2016). The blood vessels of the venous circulation in the pelvic area that communicate with the vertebral venous plexus lack valves which makes bidirectional flow possible. Through this mechanism tumour cells could directly reach the cranial veins via the vertebral venous plexus. Finally, it should be considered that the isolated cerebellar metastasis in our patient is secondary to a pulmonary or other metastasis which was not detectable on radiological imaging, or the systemic chemotherapy treated the primary and systemic metastatic lesions but not the brain metastasis due to the blood brain barrier. Therefore, repeat CT imaging of the thoracic and abdominal regions may not reveal any primary distant metastases in this case after treatment, although pre-treatment imaging did not demonstrate any systemic metastases either.

In cases with isolated metastasis at an uncommon location without other metastatic sites, examination through PET may have additional value in detecting distant metastases that were not discovered on CT imaging. Several studies have reported sensitivities between 50 and 100% and specificities of 86–100% for FDG PET/CT to detect metastases outside the pelvic area from UC (Bouchelouche, 2022). Furthermore, in two studies FDG PET/CT was shown to be superior to conventional CT staging with sensitivities of 54% and 74.9% vs. 41% and 43.7%, respectively, and two other studies demonstrated that findings from FDG PET/CT may influence diagnostic and treatment strategy in up to 68% of patients (Goodfellow et al., 2014; Kim, 2020; Apolo et al., 2010; Kibel et al., 2009). In addition, FDG PET/CT may be useful in detection of recurrent muscle-invasive UC (Bouchelouche, 2022). However, these studies did not specifically focus on the detection of thoracic or abdominal metastases in patients with a known isolated CNS metastasis, and, hence, the use of FDG PET in the diagnostic work-up of this patient group requires more research. The patient from our case report did not undergo PET examination as he reported no neurological symptoms at the time of the diagnosis of the primary tumour and, thus, there was no suspicion for brain metastasis in the absence of other metastatic sites. Since sequential thoracic and abdominal CT imaging at 4, 8 and 18 months demonstrated no growth of metastatic lesions either and the choice of treatment for the CNS metastasis would not be affected, additional PET examination was not performed.

In this case report a rare case of cerebellar metastasis originating from UC is described in detail. Several hypotheses have been proposed that may explain the absence of other distant metastases and the interventions performed in this patient are carefully evaluated. Furthermore, a comprehensive overview of literature is provided covering cases with CNS metastasis from UC based on histological confirmation. However, this case report is limited by the paucity of literature on this patient group and trials assessing therapies for patients with CNS metastasis from UC.

Isolated cerebellar metastasis from urothelial carcinoma without other distant metastases is a rare phenomenon with an uncertain route of metastatic distribution. Therefore, histologic confirmation is essential to confirm the type of primary tumour, particularly when there is a longer time interval between detection of the primary tumour and brain metastasis. Moreover, PET CT may have additional value to exclude other distant metastases. Although survival after UC is improving, the prognosis of this disease with intracranial metastasis remains poor. For our patient, treatment of UC appears to have been successful at 18 months follow-up. However, due to the symptomatic recurrence of the cerebellar metastasis the prognosis in this patient is unfavourable.

## Funding

This research did not receive any specific grant from funding agencies in the public, commercial, or not-for-profit sectors.

## Ethics approval

Not applicable.

## Consent to participate

Not applicable.

## Written consent for publication

Consent for publication was received from the patient described in the manuscript by his treating physician.

## Availability of data and material

Not applicable.

## Authors' contributions

All authors were involved in the treatment of the patient, analysis and interpretation of diagnostic data described in this manuscript. EJAV and HR drafted the manuscript. HR, WT, RNT and MCAG revised the manuscript. All authors have approved the current form of this manuscript for submission.

## Declaration of competing interest

The authors declare that they have no known competing financial interests or personal relationships that could have appeared to influence the work reported in this paper.
